# Hepatic myeloid-derived suppressor cells in tumor bearing mice exacerbate hepatitis and transform into pro-inflammatory myeloid cells

**DOI:** 10.1186/2051-1426-1-S1-P166

**Published:** 2013-11-07

**Authors:** Jose Medina-Echeverz, Tamar Kapanadze, Chi Ma, Austin Duffy, Jaba Gamrekelashvili, Veena Kapoor, Jonathan M Weiss, Robert H Wiltrout, Jay Berzofsky, Masaki Terabe, Michael P Manns, Francesco M Marincola, Ena Wang, Firouzeh Korangy, Tim F Greten

**Affiliations:** 1 GI-Malignancy Section, Medical Oncology Branch, National Cancer Institute, National Institutes of Health, Bethesda, MD, USA; 2 Department of Gastroenterology, Hepatology and Endocrinology, Hannover Medical School, Hannover, Germany; 3 Hematology Branch, National Heart, Lung, and Blood Institute, National Institutes of Health, Bethesda, MD, USA; 4 Laboratory of Experimental Immunology, National Cancer Institute, National Institutes of Health, Bethesda, MD, USA; 5 Vaccine Branch, National Cancer Institute, National Institutes of Health, Bethesda, MD, USA; 6 Infectious Disease and Immunogenetics Section, Department of Transfusion Medicine, Clinical Center and trans-NIH Center for Human Immunology, NIH, Bethesda, MD, USA

## 

Myeloid-derived suppressor cells (MDSC) represent a heterogeneous population of immature myeloid cells that accumulate in blood, liver, spleen and tumors upon chronic inflammation and tumor development in patients and mice. Acute hepatitis is characterized by a fast infiltration of inflammatory cells in the liver and increased enzymatic activity at this organ that could lead into liver fibrosis and chirrosis. We have studied the biology of hepatic MDSC in acute hepatitis. Unexpectedly, hepatic MDSC, which accumulate in the liver of mice bearing subcutaneous tumors, failed to suppress inflammatory responses upon Con A injection, but instead were responsible for exacerbating acute liver damage. Phenotypic, genetic and functional studies demonstrated rapid changes of hepatic MDSC from a suppressor phenotype into a pro-inflammatory subset as early as 3 hours after Con A injection. An increase in the expression of pro-inflammatory cytokines, costimulatory molecules such as CD80, CD86 and CD40 along with a loss of suppressor function was noticed in mice upon Con A treatment. These changes were CD40-dependent and not found in CD40-/- MDSC. Interestingly, CD40 ligation of human MDSC in vitro resulted in down-regulation of arginase I expression and suppressor function. Finally, blockade of ROS production in hepatic MDSC ameliorated hepatocyte damage suggesting that MDSC mediated toxicity was ROS dependent. We believe that these findings reflect how MDSC plasticity can be modulated to promote inflammation, opening a new path for therapies targeting innate suppressive cells in cancer patients.

